# Interdisciplinary approach of Yalom's group therapy factors: A theoretical model for including animal presence in social work education and practice

**DOI:** 10.3389/fvets.2022.1024355

**Published:** 2022-10-12

**Authors:** Alina Simona Rusu, Rebecca Davis

**Affiliations:** ^1^Faculty of Animal Sciences and Biotechnologies, Civic Engagement Center, University of Agricultural Sciences and Veterinary Medicine, Cluj-Napoca, Romania; ^2^Office of Global Programs, School of Social Work, Rutgers, The State University of New Jersey, New Brunswick, NJ, United States

**Keywords:** group therapy, human-animal interactions, education, social work, qualitative analysis

## Abstract

An increasing number of studies in the field of Social Work (SW) address the incorporation of animal presence in practice and improved understanding of social support and therapeutic components. Education programs on the ethical and practical principles of animal-assisted interventions, including models and theories supporting the psycho-physiological effects of human-animal interactions (HAI), are being implemented around the world, especially in the US. While designing and implementing a new interdisciplinary curriculum can be time-consuming and, depending on the many variables, integrating elements of HAI components in existing curricula can be a more efficient approach. We present a step-by-step approach for inclusion of HAI knowledge and practice in teaching Yalom's principles and therapeutic factors of group therapy to SW students. Based on a qualitative analysis of the existent literature and on the results of several research projects in the field of HAI, we propose an approach for infusing research-informed examples and theories supporting the beneficial effects of HAI in the direction of the interdisciplinary understanding of the Yalom's primary factors in the therapeutic process, such as: instillation of hope, corrective recapitulation of the primary family group, development of socializing techniques, imitative behavior, interpersonal learning, and group cohesion. Applied values of HAI are discussed for each factor, emphasizing the added value of animal presence in group therapy settings from the perspective of the dynamic of interspecific social networks, i.e., animal-handler-group members.

## Introduction

An increasing number of studies in the field of Social Work (SW) address the incorporation of animal presence in education and practice, in the direction of improving the understanding of the social support components. Education programs that familiarize students with ethical and practical principles of animal-assisted interventions (AAI), including models and theories supporting the psycho-physiological effects of positive human-animal interactions (HAI), such as interactions with companion, emotional support and therapy animals, have started to function around the world.

Animals represent a significant presence in the history of human lives (families, homes, communities, societies). The increasing number of programs and scientific publications in the field of human-animal bond stem from the fact that knowledge and awareness about HAIs can offer important insights to the understanding and adjust the functionality of social systems. The potential mechanisms of change behind the benefits of HAIs on human mental and social wellbeing can motivate Social Work professionals and students to consider their inclusion in their practical approach to addressing problematic functioning of individuals in various interactional contexts, both intraspecific ones (interpersonal human-human interactions) and interspecific ones (human-animal interactions). This assumption is supported by the emergence of fields of SW practice addressing aspects of HAI have emerged in the last decade in the U.S., such as *animal-assisted social work* (1–3) and *veterinary social work* (3).

As indicated by several authors and practitioners combining AAI with clinical psychology and clinical SW, the most prevalent arguments for the integration of HAIs, especially of the relationships with companion animals, into SW education and practice are: (1) animals are part of family systems/human ecologies; (2) there is a growing evidence base for the links between animal cruelty and forms of family dysfunction and criminal behavior; and (3) companion animals add therapeutic value to interventions across diverse age and special needs categories (2, 3).

In a previous study (4), we performed a qualitative analysis of the existent literature on civic engagement (Service-Learning) programs for Social Work students in connection to animal-assisted activities in order to identify the elements that could be used for the development of an interdisciplinary curriculum addressing the civic development of students through the inclusion of animal presence in education and community practice. The proposed learning objectives for the above-mentioned interdisciplinary curriculum were the following (4):

- Identify and apply theories of human behavior and principles of ecological social work animal-assisted interventions with individuals, families, and groups;
- Demonstrate empathy, reflection, and interpersonal skills through the connection between HAI and civic engagement activities;
- Use and translate research evidence on animal inclusion in social work practice to inform and improve practice;
- Demonstrate engagement, assessment, and intervention practice skills through positive human-animal interactions based activities.

The aim of our current study is to provide a transversal learning objective as an addition to the four learning objectives above, by introducing SW students and practitioners interested in the AAI field to a research-informed body of work coming from the field of group work and group therapy. This transversal learning objective refers to Yalom's eleven (5) therapeutic factors of group therapy (6). This framework is proposed in order to facilitate a better understanding of the mechanisms of change related to the benefits that animal presence can bring to group interventions.

The proposed transversal learning objectives informed by a group therapeutic intervention is important because all of the current explanatory theories and models behind the benefits of HAIs on aspects of human life are based on the social nature of interactions, i.e., meaningful social encounters with others (humans and animals). Examples of theories that are commonly found in the SW practice literature supporting the inclusion of HAI (1, 2, 7, 8) are: ecological-systems theory (9), family theory and family-centered practice (10), social support theory (11), and the strengths perspective (5).

Theories from the field of ethology and evolutionary sciences are often found to contribute to the theoretical explanatory frame of the psychological, social and physiological benefits of positive HAI (e.g., stress reduction, cognitive activation, enhancement of social skills, decrease of performance anxiety, decrease of social isolation, decrease of probability of cardio-vascular diseases etc.). Most of these theories emphasize the intrinsic qualities of animals as social agents, especially of companion animals and their availability for interaction and acceptance of proximity, which are often related to a calming effect and expression of positive affect in humans (12, 13). The *biopsychosocial model of health* supports an integrative conceptual frame for the identified theories in regards to the benefits that animals can bring to several aspects of human quality of life (13, 14). This model argues that health can be seen as the result of a combination of biological, psychological, and social factors that are interrelated, with animals being a significant component of human ecologies. Incorporating ecological-systems theory (9), there are reciprocal benefits (at levels of survival and social functioning) that humans and animals bring to each other.

Group therapy is a therapeutic method applied to clinical social work practice in which individuals are placed in a group, guided by one or more therapists or leaders for the purpose of bringing about change(s) in each individual through the various group processes. Unique to the group is that it allows for the re-creation of group members' customary roles, behaviors, and interactional patterns (6). The power of groups is that social workers are required to bring the underlying issues and feelings out in the open in a relatively “public way” (15), while building cohesion experienced as togetherness and connection (6). This process can be challenging for groups, especially when dealing with personal topics such as intimate relationships, problem-behaviors, parenting behaviors, and work and career-related issues, etc. (6). Group therapy focuses on the “here and now” (6), utilizing action-oriented problem-solving and decision-making that values a range of activities including art, music, games, theater, etc. (15). This theoretical underpinning supports the introduction of objects, both animate and inanimate, such as dogs, animals into the therapeutic process that has the potential for mutually shared and reciprocal benefits.

The change process in groups refers to the dynamics of the group and the emotional and cognitive processes within each individual, that foster social and emotional functioning and more effective coping abilities. Irvin Yalom, an American existential psychiatrist and currently an emeritus professor of psychiatry at Stanford University, states “therapeutic change is an enormously complex process that occurs through an intricate interplay of human experiences,” which he refers to as “therapeutic factors,” initially termed “curative factors” [5, p. 1]. Yalom's eleven group therapy factors, although examined separately, are “intricately interwoven” and vary in their importance from group to group [5, p. 2]. The eleven therapeutic factors briefly described here include:

1. *Instillation of Hope*: A belief things can be better; positive expectations;
2. *Universality*: “all in the same boat” phenomenon; “misery loves company”;
3. *Imparting Information*: Didactic instruction; advice and direct guidance;
4. *Altruism*: Giving to others; group therapy is unique as it offers members the opportunity to benefit themselves as well as others;
5. *Corrective Recapitulation of the Primary Family Group*: Group resemblance of one's primary family group with authority figures, siblings, strong emotions, intimacy, etc.;
6. *Development of Socializing Techniques*: Learning and testing new ways to interact and engage with each other;
7. *Imitative Behaviors*: Learning by observing other members interacting, solve problems, etc.
8. *Interpersonal Learning*: Learning and experiencing the importance of the human relationships and the reciprocity of secure attachments;
9. *Group Cohesiveness*: The “we-ness” of group therapy; level of solidarity among and between members—a critical group therapy factor;
10. *Catharsis*: Discharge of strong emotions;
11. *Existential Factors*: Transcending suffering and realities of life through faith, relationship, and hopefulness found through something or someone else (6).

Joyce et al. (16) reduce these eleven factors to four more global factors, which include: instillation of hope, secure emotional expression, awareness of relational impact, and social learning.

## Methods

The aim of this methodological approach is to provide an example of how SW students and practitioners can identify and apply Yalom's group therapeutic factors to AAI-group intervention studies published in peer review journals (i.e., studies that provided enough data for the calculation of the effect size of the interventions).

This methodological exercise is proposed within the transversal learning objective and it aims to answer the following **research questions**:

- What type of explanatory theories are the authors of the AAI-group based studies providing on the justification of using a group form of intervention?
- Can one identify elements corresponding to Yalom's group therapeutic factors in the text provided by the authors of the AAI-group studies?

According to Erlingson and Brysiewicz (17), the objective of a qualitative content analysis is to systematically transform a large amount of text, considered the raw data, into a concise and organized summary of the key results. The following steps were performed: (1) Reading the written reflections to gain a general understanding of what the participants are expressing; (2) Condensation of the text by division into meaning units (codes); (3) Grouping the codes into categories (i.e., a category is formed by grouping together those codes that are related to each other through their content or context) and themes (i.e., a theme can be seen as expressing an underlying meaning found in two or more categories).

For this study, the thematic qualitative analysis was performed on the *Introduction* and *Discussion parts* of the four publications included in the systematic review performed by O'Haire et al. (18) on empirical studies of AAI for trauma. The ***predefined themes*** in the qualitative analysis are Yalom's 11 therapeutic factors (6). The text was manually condensed by ASR, followed by discussion and agreement between the two authors regarding the codes.

Among the ten studies qualified for inclusion (six peer-reviewed journal articles and four unpublished theses) in the systematic review performed by O'Haire et al. (18), various forms of **group intervention** were used in five studies, either alone or combined with individual therapy. From the five group intervention studies, the four published scientific papers with full access were included in this analysis (four papers, see Table 1). The fifth study not included in our analysis was an unpublished dissertation thesis. The four studies using AAI-group interventions and with sufficient data to calculate the effect size of the interventions in the meta-analysis research articles (18) are presented in Table 1.

**Table 1:** The characteristics of the four AAI-group intervention studies included in the meta-analysis of O'Haire et al. (18).

**Title and code of the AAI-group intervention studies**	**Characteristics of the study (18)**
**Study 1 (S1, 19):** Balluerka, N., Muela, A., Amiano, N., and Caldentey, M. A. (2014). Influence of animal-assisted therapy (AAT) on the attachment representations of youth in residential care.	• Age of participants (12–17 y); Format: individual and group therapy; Animal type: dog, horse, cat, farm animals; Duration: 12 weeks, two-three sessions/ week. • Outcomes: increase of attachment security. • Effect size (d) pre-AAI vs post-AAI: not enough data for calculation of the effect of the intervention.
**Study 2 (S2, 20):** Dietz, T. J., Davis, D., and Pennings, J. (2012). Evaluating animal-assisted therapy in group treatment for child sexual abuse.	• Age of participants (5, 8–17); Format: group therapy; Animal type: dog; Duration: 7 weeks, one-two session/ week. • Outcomes: decrease of PTSD symptoms, decrease of depressive symptoms, decrease of anxiety, dissociation and anger, decrease of sexual concerns. • Effect size (d) pre-AAI vs post-AAI: for depressive symptoms (d = 0.92, *p* < 0.001), for PTSD symptoms (d = 0.86, *p* < 0.001), for anxiety (d = 0.80, *p* < 0.001).
**Study 3 (S3, 21):** Hamama, L., Hamama-Raz, Y., Dagan, K., Greenfeld, H., Rubinstein, C., and Ben-Ezra, M. (2011). A preliminary study of group intervention along with basic canine training among traumatized teenagers: a 3-month longitudinal study.	• Age of participants (14–16 y); Format: group therapy; Animal type: dog; Duration: 12 weeks, one session/ week. • Outcomes: decrease of PTSD symptoms, decrease of depressive symptoms, increase of subjective well-being, increase of coping abilities. • Effect size (d) pre-AAI vs post-AAI: for depressive symptoms (d = 0.47, *p* = 0.06), for PTSD symptoms (d = 0.7, *p* < 0.05).
**Study 4 (S4, 22):** Kemp, K., Signal, T., Botros, H., Taylor, N., and Prentice, K. (2014). Equine facilitated therapy with children and adolescents who have been sexually abused: a program evaluation study.	• Age of participants (5, 9–17); Format: group therapy; Animal type: horse. Duration: 9–10 weeks, one session/ week. • Outcomes: decrease of depressive symptoms, decrease of maladaptive behavior, decrease of PTSD symptoms, decrease of anxiety, dissociation and of sexual concerns. • Effect size (d) pre-AAI vs post-AAI: for depressive symptoms (d = 1.31, *p* < 0.001), for PTSD symptoms (d = 3.77, *p* < 0.001), for anxiety (d = 2.63, *p* < 0.001).

## Results

As indicated by the data summarized in the systematic review (18), the participants in the four AAI-group interventions were predominantly survivors of child abuse. The most common animal species were dogs and horses. The activities were described mainly in relation to the animal presence, with no details on the dynamic of the interactions among the group members during the therapy program. All of the interventions had duration of from 7 to 12 weeks, which would have been adequate time for observations of the group process and interactions (Table 1).

Three of the four AAI-group intervention studies reported having a predetermined theme for each session. Among the activities described, two main variation factors were identified: the animal species and whether or not the intervention animal was used as a metaphor for the child's relationship with their usual social partners (18). Two studies with dogs integrated them into the regular therapy sessions, which included both dog-focused activities such as training as well as talking to the dog about personal traumatic experiences (19, 20). In one of the studies, the integration of the dog was done through stories told from the animal's perspective (19). In this case, the effects of the animal presence were generally enhanced by telling a therapeutic story about the dog, attributed to giving the dog a social role and integrated purpose in the therapy session (18). This interpretation is supported by the efficiency of the AAI-group intervention program in this particular study using the therapeutic stories about the dog (S2), as indicated by the significant effect size values calculated for the following pre- and post-intervention outcomes: PTSD symptoms, depressive symptoms and anxiety [(18), Table 1].

The information provided by O'Haire et al. (18) indicates that the effect size was significant for the studies S2, S3 and S4 (pre- and post-intervention), i.e., the AAI-group intervention had a significant effect on decreasing the PTSD symptoms and the anxiety levels. No information on collection and analysis of qualitative data is provided in the four AAI-group intervention studies. The results of the qualitative content analysis of the *Introduction* and *Discussion* parts of the four studies are presented in Table 2.

**Table 2:** Codes identified within the predefined themes (Yalom's therapeutic factors) in the four animal-assisted group intervention studies.

**Group therapeutic factor—themes**	**Definition based on Yalom and Leszcz (6)**	**Information units (codes) identified in the four AAI-group studies (18)**
1. Instillation of hope	Process by which hope is inspired through observation of therapeutically advanced group members; provides group members with encouragement that change is possible.	• **S1** “The intervention ended by **helping participants to think, feel, and act in new ways** that were different to those they had used in past relationships. In order to achieve this goal, it was pointed out that **their current relationships could be modified positively** by **building secure relationships** involving **synchrony, reciprocity, trust and mutual support, empathy, and sincerity**, relationships of the kind that they had been able to develop with the animal and the therapist.”
2. Universality	Process by which group members feel less isolated in their pain as they connect to others with similar experiences; group members learn that he/she is not alone or unique in problems and suffering.	• **S4** “Studies have shown that animals are capable of **providing unconditional positive regard without judgment**, something that may not be present in the lives of abused children (21, 22).”
3. Imparting of information	Group members receive psychoeducation/ didactic instructions and/or advice from the group leader and/or the other group members.	• **S1** “The aim of the first individual sessions was to enable participants to become familiar with the different animals and the professionals involved, and also to **establish the framework and the rules of conduct**; these latter aspects bring **constancy and predictability to the therapeutic process**” • **S1** “Participants were **guided to reshape the relational style of their interpersonal interactions** (with friends, partners), with attention being paid to different contexts of social interaction, such as the residential care setting (key worker) and school (teachers), in which they could establish new potential attachment relationships. • **S2** “Group therapy **provides children a safe environment** in which they can **receive guidance from therapists while supporting one another as they share and process their experiences** (23, 24). The group setting allows children to develop trust, which has often been eroded by the abuse usually experienced from a trusted individual (23).”
4. Altruism	Group members offer support, reassurance, suggestions and insight to other group members; group members learn that their contributions to the group are significant to the healing of others.	• **S2** “Sharing these feelings with or about the animal can **initiate the emotional sharing process** with the therapist. For the client, the **animal is seen as a friend** and ally, thus presenting a **safe atmosphere for sharing**. Using an animal **offers nurturance** through a presentation of **unconditional acceptance and interaction**.” • **S4** “The arguably innate human interest and desire to interact with animals affords animals the ability to gain a young client's attention (25), as well as **promote pro-social, humane behaviors and empathy toward others**.”
5. Corrective recapitulation of the primary family group	Group member relieves early familiar conflicts in group in a corrective, more satisfying way; the group is experienced as a familial unit allowing for relearning of unhealthy patterns learned within the family of origin.	• **S1** “The participants' past and present r**elationships were explored, including their expectations, feelings, and behaviors**. In this process they were **encouraged to reflect** upon the ways in which they engaged in **relationships with significant figures in their current life**, what their **expectations were regarding their own feelings and behavior and those of other people**…(26).” • **S1** “…we sought to help **participants become aware of how their current relationship experiences** may be closely related to events and situations faced during childhood (26)….Through these exercises the **participant is confronted with his/her own relational strategies**, which are also closely connected with his/ her upbringing and the current relational parenting style he or she is experiencing.” • **S4** “Other activities are designed to create a **metaphor between what occurs in the arena and the participant's every-day life** and again are performed at liberty. What emerges during these activities are **patterns of thinking, reactions/responses** to different situations and outcomes, and r**eactions to dynamics within the family group or within the group of participants**.”
6. Development of socialization techniques	The group provides members with an environment that fosters adaptive and effective communication and enhanced social skills.	• **S1** “In this context, the therapist and the animal function as therapeutic tools whose purpose is to **maximize the ability of the young person to develop good attachment relationships**, not only within the therapeutic triangle (therapist–client–animal) but also with his/her current caregivers from the residential care setting, with his/her friends, and in the emerging romantic relationships...”
		• **S2** “The creative process of using canines along with group intervention seemed like **enhancing their sense of confidence and belief in their abilities**, **regaining them with a sense of control and ego-mastery**.” • **S4 “**Animals have also been known to p**rovide an important emotional bridge to therapeutic alliance**, as a difficult to engage child may find it easier to engage with an animal and eventually transfer this alliance to the therapist (27).
7. Imitative behavior	Group members learn to model behaviors from other group members.	• **S2** “The experience of a client interacting with an animal can **provide knowledge about boundaries and limit setting by observing and imitating the therapist–animal interactions**.”
8. Interpersonal learning	Group members learn about their own maladaptive interpersonal patterns through feedback provided by other members; Members provide an environment that allows interpersonal interactions in a more adaptive manner.	• **S1** “The objective of the first group sessions was **to create a social microcosm of security** within which the group members **could act naturally**, a microcosm that would also serve for the **analysis of their interpersonal behavior in the social world outside the group** (28).”
9. Group cohesiveness	Group member feels connection and solidarity with other group members and the group as a whole.	• **S4 “**The arguably **innate human interest and desire to interact with animals** affords animals the ability to gain a young client's attention (25), as well as **promote pro-social, humane behaviors and empathy toward others**.”
10. Catharsis	Group members release of strong feelings about past and/or present experiences.	• **S1** “Storytelling as a part of the therapeutic process has been used to assist children in **expressing themselves**, particularly on difficult topics;” [...]the use of animals in working with child sex abuse survivors helps “them **disclose the abuse and express their feelings by projecting them onto the animal**.”
11. Existential factors	Members accept responsibility for life decisions; group members confront issues on the ultimate concerns of existence: death, isolation, freedom, and meaninglesness.	• **S1** “The intervention ended by **helping participants to think, feel, and act in new ways** that were **different to those they had used in past relationships**. In order to achieve this goal, it was pointed out that **their current relationships could be modified positively by building secure relationships** involving synchrony, reciprocity, trust and mutual support, empathy, and sincerity, relationships of the kind that they had been able to develop with the animal and the therapist.”

## Discussion

The aim of this study was to provide an example of how SW students and practitioners could apply the analytical framework offered by Yalom's group therapeutic factors for the purpose of clinical application of relevant factors in AAI-group interventions. This analysis used existing peer-reviewed studies that provided enough data for the calculation of the effect size of the interventions. Experts in the field of human-animal bond indicate that the beneficial contribution of animals' presence and positive HAI on social capital enhances individual mental and physical health, as well as the interpersonal relationship and cooperation in families, groups (including therapy groups), and communities [e.g., (4, 7)].

This methodological approach provides an analytical framework for SW students and practitioners to enhance understanding of change mechanisms related to the benefits of positive animals' presence in interpersonal contexts and the potential applications in clinical settings. This analysis was based on Yalom's group therapeutic factors as themes for a qualitative content analysis of the *Introduction* and *Discussion* sections of four group intervention studies on the effects of AAI interventions on trauma (18).

A discussion of the findings of the qualitative analysis follows that includes reflections on each of the units of analysis (coded group therapy themes) and recommendations for the facilitation of the inclusion of Yalom's cluster of factors in planning and evaluation of the AAI-group interventions programs. Dietz et al. (19) argues that AAI is not a stand-alone theory, but “augments existing treatment strategies” (p. 667) in which animals contribute to meeting the therapeutic goals.

### Instillation of hope

According to Yalom and Leszcz (6), the therapeutic factor *hope* operates differently in the group format through the emphasis on progress of others, i.e., a group member can have the insight that “*others are living inspiration*.” This inspirational side of the other members of the group can be revealed by the ways they interact with the therapy animal. Another operational aspect of hope is the mobilization through encouragement, which fosters the development of the “can do” approach. In our qualitative analysis (Table 2), the factor “instillation of hope” was supported by the codes identified in one of the four studies (S1): participants were helped to feel, think and act in new ways by pointing out that their current interpersonal relationships could be modified positively. These codes can also be included in the theme “Imparting information,” due to the fact that the information about the expectation of the positive change was communicated by the therapists involved in the group intervention.

As a recommendation to SW students and practitioners, we consider that the factor *instillation of hope* can be activated by the inclusion of animal presence in group therapy by creating opportunities for animals to assist the group members in doing activities (individually and/or together with other members), in facilitating relationships and shared experiences, and in feeling and experiencing something positive and new. Reflection during and after each activity can help in the direction of creating awareness that animals (and the other group members interacting with the animals) *can help one feel hopeful* that things can be different and that one can experience positive feelings again.

### Universality

This group therapy factor refers to the fact that individuals enter treatment feeling they are unique (6), often due to extreme social isolation. In a group, learning that “*we are all in the same boat”* can be a powerful source of relief, at least in the early stages of group formation. The qualitative analysis allowed us to identify codes related to this factor in one of the four AAI-group studies (Table 2), in which the authors cited two studies from the AAI literature .”..*animals are capable of providing unconditional positive regard without judgment”* [(21, 22) *cited in* (20)].

For a better understanding and usage of the therapeutic factor “universality,” as well as other Yalom's group therapy factors, such as “group cohesiveness,” and “existential factors,” SW students and practitioners are recommended to become aware of some of the theories and research-informed models behind the idea that animal presence in group therapy can facilitate the interpersonal and human-animal connections through petting and playing with the animal. These play-based activities have the potential to foster feeling and sharing affection and enjoyment in the presence of the animal's unconditional love. As pointed out by Yalom and Leszcz (6), sharing the universal human condition of wanting and needing positive attention and care can be a big step in dealing with shame, guilt, stigma etc.

The effect of a friendly animal on interpersonal interactions is known as *the social catalyst effect* (29). Also, HAI research indicates that direct contact with life forms other than humans appear to have a modulatory effect on the psychological and physiological parameters associated with social interactions [e.g., (29)]. The presence of oxytocin is considered as an optimal method for providing concrete evidence of the positive effects of HAI on human wellbeing. Oxytocin is a peptide hormone that regulates various physiological, psychological and behavioral functions in humans and animals, mainly by lowering the level of stress hormones such as cortisol [(30), cited in (31)]. Also, oxytocin production promotes positive affective states associated with interpersonal relationships, such as mother-infant bond, couple relationships and positive social interactions (30).

### Imparting information

The process of advice-giving/seeking rarely benefits group members and it needs to be managed by group leaders/ therapists. The focus of the neophyte group members tends to be on “reasoning” and usually it reflects a resistance to more intimate engagement (6). In our qualitative analysis, codes belonging to the theme “imparting information” were found in two AAI-group studies included in systematic review of O'Haire et al. (18), e.g., professionals involved in leading of the group interventions offered guidance to the participants in different phases of the group interactions (Table 2). In the case of S1, in the beginning sessions, information was offered in order to establish an appropriate level of familiarity with the animals, to establish the framework and the rules of conduct that were considered important to bringing constancy and predictability to the therapeutic process. During the following sessions, participants were guided to reshape the relational style of their interpersonal interactions (32). In the second study (S2), the authors provided references from the literature indicating the importance of group members receiving guidance from therapists while supporting one another as they share and process their experiences in order to provide participants a safe environment (19).

### Altruism

The “altruism” therapeutic factor refers to people's need to feel that they are needed and useful (6). Altruism can operate in various types of healing and acts of forgiveness that can be done together with the group members, such as preparing a feast together, performing a type of community service, etc. As pointed out by Yalom and Leszcz (6), the neophyte group members do not, at first, appreciate the healing impact of other members, so they may actively resist helping others or receiving help. This factor refers to the process in which group members offer support and guidance to other members and become aware of the healing valence of their contributions. Our qualitative analysis revealed codes corresponding to this factor in two AAI-group intervention studies (Table 2), i.e., animal is seen as a friend and ally that facilitates a safe atmosphere for sharing and initiating the emotional sharing process (S2) that promotes pro-social, humane behaviors and empathy toward others (S4).

Theories that could facilitate a deeper understanding by SW students and practitioners, as well as by the group members (in terms of psycho-education), of the mechanism of change related to altruism as a curative factor in group settings, can be found in the conceptual framework of HAI. One of the theories is the *biophilia hypothesis* (33, 34), which argues that human individuals possess an innate tendency to be attracted to and positively interact with any living organism, including plants. This theory can be also found in the explanatory framework of the positive effects of nature-assisted activities in the fields of rural social work and eco-social work (or ecological social work) (35). Another theory, which is commonly found in the HAI literature, as well as SW's evidence-base, is *attachment theory* (26). Animals are often perceived as important attachment figures, mainly due to their proximity in everyday life and by facilitating a secure affective environment as a source of social support in various interpersonal contexts [e.g., (36–38)].

### Interpersonal learning and development of socializing techniques

The “altruism” therapeutic factor is usually connected with the factors “***interpersonal*** ***learning***” and “***development of socializing techniques***.” In our qualitative analysis, the “interpersonal learning” therapeutic factor emerged in one of the AAI-group studies (S1), i.e., the objective of the first AAI group session was to create a social microcosm of security that motivates the group members to act naturally and build a reference base for the analysis of their future interpersonal interactions. Interpersonal learning is considered the basis of group therapy, in the way insight, transference, and the corrective emotional experience are considered in individual therapy (6).

The “*development of socializing techniques*” factor refers to the environment provided by the group in the direction of fostering adaptive and effective communication and enhanced social skills; the explicitness of the process varies depending on the age of group members, problems presented etc. In our qualitative analysis, codes belonging to this theme were identified in three AAI-group studies (S1, S2 and S4; Table 2). These codes referred to the following aspects: the dyad animal-therapist was as a tool for maximizing the ability of young group members to develop good attachment relationships (S1), the role of canine-assisted therapy in enhancing the sense of confidence in their abilities, including those related to sense of control and ego-mastery (S2), and the role of animals in providing an emotional bridge in therapeutic alliance and social engagement with other group members (S4). Animal presence in the context of this therapeutic factor can operate to teach the group members a series of social abilities, such as patience, waiting one's turn, observation, giving and receiving instructions, etc.

We recommend that a better understanding of these two factors by SW students and educators working in AAI-group contexts can be provided by knowledge regarding the importance of interpersonal relationships. They allow the shift from relief of suffering to change in interpersonal functioning, which is considered an essential early step in the dynamic therapeutic process. In other words, the interpersonal interactions within the group help the members to translate depression into interpersonal terms (e.g., isolation and difficulty reaching out to others). In line with this, we consider that SW students and practitioners should become aware that animal presence can be used in group contexts to transcend these learned cognitions and behaviors, by facilitating the connection with our spontaneous and nurturing “self,” i.e., if we cannot reach out to humans, we can start by reaching out to a forgiving and non-judgmental animal.

Animal presence, especially when included in functional group play situations, can help group members transcend the difficulty in expressing a need and asking for attention (spontaneous interactions). According to Leconstant and Spitz (39), interspecific play is considered a fertile venue to explore the capacity to correctly perceive and interpret signals emitted by partners (40). In terms of the mechanism of change, Porges (41) defines interactive play as a neural exercise, which requires synchronous and reciprocal behaviors between individuals as well as an awareness of the level of social engagement of each individual. This explanation coming from neurosciences and ethology supports not only the “***interpersonal learning***” and “***development of*** ***socializing techniques***” Yalom's group therapy factors, but also the “***imitative behavior***” factor.

Moreover, interspecific play can offer valuable opportunities for the **“*catharsis*”** factor to operate, i.e., group members can observe in a mindful way the play scenes between the therapy animal and others, or they can immerse themselves in the “now” moment of playing with the animal and the other members of the group. Reflection can play an important role for processing what is observed, experienced and learned.

### Imitative behavior

In line with the factors referring to interpersonal learning and social skills, this factor refers to the process by which group members learn to model behaviors from other group members (6). Codes reflecting the “imitative behavior” factor were identified in our qualitative analysis in one of the four AAI-group studies, i.e., S2, in which the authors specify that the experience of the group members interacting with an animal can provide knowledge about boundaries and limit settings by observing and imitating the interactions of the therapist-animal dyad. No information regarding the observations of other group members interacting with the animals is provided in the four AAI-group therapy studies.

We recommend the inclusion in the explanatory framework of “imitative behavior” Yalom's group therapy factor of the concept *social affordances*. Social affordances are defined as possibilities for social interaction offered by an environment (39), in this case a heterospecific one, due to the animal presence in the group setting. When operating with the social affordances concepts, various authors, see for example [(41, 42) cited in (39)] take into consideration the possibility that the intentionality of actions and the ability to understand the intentions of others are based more on primary and sensorimotor processes than on specialized cognitive abilities. When an individual is observing another individual petting and/or playing with a therapy animal in the context of a group environment, the process of resonance *via* mirror neurons can be understood as part of an intersubjective perceptual process (43).

### Corrective recapitulation of the primary family group and existential factors

The factor “*corrective recapitulation of the primary family group*” refers to the group experience as a familial unit allowing for relearning of unhealthy patterns learned and/or maintained in the family of origin. In our qualitative analysis, codes reflecting this factor were identified in two of the four AAI-group intervention studies included in the systematic review of O'Haire et al. (18). In the first study (S1), the codes included information such as: “*the participants' past and present relationships were explored, including their expectations, feelings, and behaviors*,” and “*participants become aware of how their current relationship experiences may be closely related to events and situations faced during childhood*,” while in the S4, the authors indicated that the activities with the animals allowed the emergence of “*patterns of thinking, reactions/responses to different situations and outcomes, and reactions to dynamics within the family group or within the group of participants*” (Table 2). Related to the factor mentioned above is the Yalom's group therapy factor “*existential factors*,” which refers to the group as facilitating a safe environment for the members to confront issues on concerns of existence, e.g., isolation, meaninglessness, freedom, etc. This factor emerged in our qualitative analysis in one of the AAI-group studies (S1, Table 2), i.e., “*it was pointed out to the participants that their current relationships could be modified positively by building secure relationships...relationships of the kind that they had been able to develop with the animal and the therapist.”* This code was included in the “imparting information” factor, too.

### Group cohesiveness

Even though the *Group cohesiveness* therapeutic factor was reflected by our content analysis in only one out of the four studies included in the systematic review, O'Haire et al. (18) did mention in the Introduction part that animals have been demonstrated to act as social facilitators that can connect people, thus helping individuals with PTSD to connect to other persons around them. The code reflecting this factor was attributed to the “altruism” group therapy factor, too, because it refers to the innate human interest and desire to interact with animals, which can promote nurturing behaviors and positive affects toward others (S4).

## Conclusions

Based on the findings of the qualitative analysis aiming to identify codes reflecting Yalom's group therapy factors in four evidence-based AAI-group studies, we recommend that Yalom's group therapy factors be fostered in AAI-group interventions by offering information/ psycho-education to the professionals and to the group participants about the theories supporting the beneficial effects of direct contact with the animals and about the potential mechanisms of interspecific positive interactions, i.e., the recently published *Integrative Model of Human-Animal Interactions* [IMHAI; (39)].

The IMHAI provides an interdisciplinary conceptual framework for the study of interspecies interactions (including animal-assisted interventions), which is based .”..*on a systemic approach to the study of primary-process emotional affects during interspecies social interactions, through the processes of emotional transfer, embodied communication and interactive emotional regulation*” [IMHAI; (39)]. The authors specify that emotions can be generated in social contexts and that they have an important social dimension and a high communicative value [(44), cited in (39)]. In developing the IMHAI model, the authors have approached the primary emotions according to the individual's perception of the physical and social environment in which the interactions are taking place. In social species, interactions with familiar individuals, that can be peers or family members (in this case, the other group members), are critically important to the healthy development and functionality of individuals (45, 46).

We consider that the interactionist approach to interspecies social phenomena offered by the IMHAI model represents an excellent connecting element of Yalom's group therapy model with the other explanatory theories of the beneficial effects of animal-assisted interventions, not only in terms of better understanding the mechanism of change, but also in terms of documenting and planning animal-related engagement (ARE) group interventions. Through this interactionist approach, SW students and practitioners can gain a valuable point of view where, according to Leconstant and Spitz (39), the basic unit of social analysis is not the individual action, but rather the system, in this case a heterospecific social system, formed by the set of actions that occur between individuals. The ways in which individuals respond to each other to generate a situation can be observed and analyzed from the perspective of Yalom's therapeutic factors.

It is important to mention that the authors of IMHAI point out that the communication process is considered “*as a global phenomenon and integrates all patterns of behavior that can have a communicative value, such as speech, facial expressions, gaze, gestures, interpersonal distance, etc*.” (39). Therefore, knowledge on the species-specific behavior of the animals included in AAI (e.g., type of social organization, body language, pacifying behaviors, etc.) is important to be considered in developing an interdisciplinary curriculum for the incorporation of animal presence in the education and practice of Social Work students. Moreover, any conceptual model referring to the mechanism of change in the area of animal-assisted interventions should include considerations of the positive and negative effects of the interspecific interactions on both humans and animals (2, 47), with clear references to investigations of the behavioral indicators of the wellbeing of animals included in AAI, as well as on the ethical standards associated with AAI in social work [e.g., (3, 48)].

A particular type of animal-assisted group intervention in which Yalom's therapeutic factors could serve as a guiding framework for SW professionals involved in the implementation of the interventions is represented by prison-based animal programs. The inclusion of animal presence in group therapy in criminal justice settings might foster a climate of social support among inmates and prison staff. In line with this, the interpretation offered by Allison and Ramaswamy (49) regarding the potential of AAI to strengthen the intersubjectivity between inmates and prison staff, including social workers, reflects some of the Yalom's therapy factors, such as hope, connectedness and interpersonal learning. The authors recommend that future studies on animal-assisted group therapy in prison settings should pay attention to standardizing procedures to measure empathetic alliances, connections to the therapeutic content and other motivational aspects related to the therapy process (49). These procedures could include questionnaires allowing the identification of the Yalom's therapeutic factors [e.g., (16, 50)].

## Data availability statement

The original contributions presented in the study are included in the article/supplementary material, further inquiries can be directed to the corresponding author/s.

## Author contributions

All authors listed have made a substantial, direct, and intellectual contribution to the work and approved it for publication.

## Conflict of interest

The authors declare that the research was conducted in the absence of any commercial or financial relationships that could be construed as a potential conflict of interest.

## Publisher's note

All claims expressed in this article are solely those of the authors and do not necessarily represent those of their affiliated organizations, or those of the publisher, the editors and the reviewers. Any product that may be evaluated in this article, or claim that may be made by its manufacturer, is not guaranteed or endorsed by the publisher.
